# Pharmacokinetic Study of NADPH Oxidase Inhibitor Ewha-18278, a Pyrazole Derivative

**DOI:** 10.3390/pharmaceutics11090482

**Published:** 2019-09-17

**Authors:** Seul Gee Lee, Jaeok Lee, Kyung Min Kim, Kee-In Lee, Yun Soo Bae, Hwa Jeong Lee

**Affiliations:** 1 Graduate School of Pharmaceutical Sciences, Ewha Womans University, Seoul 03760, Korea; ysg0483@naver.com (S.G.L.); leejo19@ewha.ac.kr (J.L.); 2 Graduate School of Industrial Pharmaceutical Science, Ewha Womans University, Seoul 03760, Korea; kkm951106@naver.com; 3 Korea Research Institute of Chemical Technology, Daejeon 34114, Korea; kilee@krict.re.kr; 4 Department of Life Science, Ewha Womans University, Seoul 03760, Korea; baeys@ewha.ac.kr

**Keywords:** pharmacokinetics, human applicable formulation, pyrazole derivative, NOX1/2/4 inhibitor, osteoporosis

## Abstract

In a previous study, the specific NOX1/2/4 inhibitor Ewha-18278 was confirmed as a possible treatment for osteoporosis both in vitro and in vivo. Here, we investigated the pharmacokinetics (PK) of the compound by intravenous (IV) and oral administrations to rats. Dimethyl sulfoxide (DMSO)-based and diazepam injection-based formulations were used to dissolve the compound. In the latter formulation applicable to humans, the changes in PK parameters were monitored at two different concentrations (1 mg/mL and 2 mg/mL). The area under the plasma concentration-time curve from zero time to infinity (AUC_inf_) of Ewha-18278 was highest in the DMSO-based formulation (2 mg/mL). Also, the concentration was increased 1.6-fold at the low concentration of the diazepam injection-based formulation compared to the high concentration. There was no statistical significance in the AUC_inf_ of the compound between DMSO-based formulation (2 mg/mL) and diazepam injection-based formulation (1 mg/mL). These results suggest that Ewha-18278 can be delivered to humans by both IV and oral routes. In addition, the diazepam injection-based formulation of Ewha-18278 appears to be a suitable candidate for dosage development for future toxicity test and clinical trial.

## 1. Introduction

NADPH oxidases (NOX enzymes), widely distributed in a variety of tissues, induce oxidative damage in various pathologic conditions including osteoporosis [1]. Among seven NOX members, NOX1, 2, and 4 play crucial roles in osteoporosis [2]. The NOX enzymes involved in receptor activator of NF-κB ligand (RANKL)-dependent osteoclast cell differentiation induced reactive oxygen species (ROS) production as a part of the pathogenesis [3,4]. Therefore, the inhibition of NOX-mediated ROS production has provoked interest as a therapeutic strategy for diseases. More selective targeting of the specific NOX enzymes will be helpful in increasing the therapeutic potency and decreasing toxicity.

The novel pyrazole derivative Ewha-18278 was developed to selectively inhibit NOX1, NOX2, and NOX4 to treat osteoporosis [5]. The pyrazole derivative inhibited the responses of bone marrow-derived macrophages (BMMs) to RANKL as well as directly interfering with the activity of NOX1/2/4 in vitro [5]. Moreover, the anti-osteoporotic function of the derivative was confirmed in vivo. Ewha-18278 induced bone strength by increasing cortical bone thickness in estrogen-deficient ovariectomized (OVX) mice when Ewha-18278 was orally delivered at 10–20 mg/kg daily for 4 weeks [5]. However, its clinical uses have not been investigated yet.

Pharmacokinetic (PK) study provides necessary information on drug absorption, distribution and elimination [6]. Based on PK profile, the dosage regimen of a certain drug to treat the disease can be determined. In addition, the development of an appropriate PK model is important to predict precisely the drug absorption, distribution, elimination, and/or bioavailability (BA) after the administration. 

Here we investigated for the first time the PK of Ewha-18278, the NOX inhibitor in rats, as part of the preclinical process of drug development. To determine plasma concentrations of Ewha-18278, a simple HPLC/UV analytical method was developed and validated. For PK study, a dimethyl sulfoxide (DMSO)-based formulation suitable for animals and a diazepam injection-based formulation applicable to humans were used to obtain PK parameters of the NOX inhibitor. The results were compared after intravenous (IV) and oral administrations. Moreover, PK parameters of the compound were compared between two different concentrations, 1 mg/mL and 2 mg/mL, of the formulation applicable to humans, the diazepam injection-based formulation.

## 2. Materials and Methods

### 2.1. Materials

**Figure 1:**
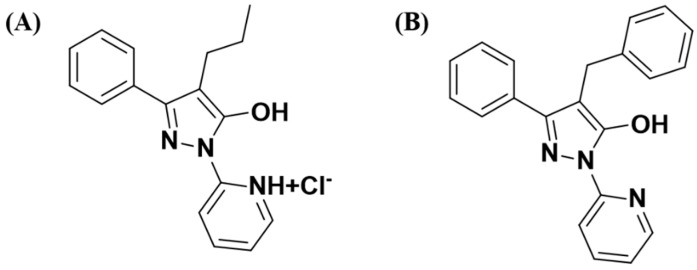
The chemical structures of (**A**) Ewha-18278 (NOX inhibitor) and (**B**) Ewha-18212 (Internal standard (IS)).

NOX inhibitor (Ewha-18278) and internal standard (IS, Ewha-18212) (Figure 1) were synthesized as reported previously [5]. DMSO, Cremophor^®^ EL, Tween^®^ 80, and acetonitrile (ACN) were purchased from Sigma-Aldrich (St. Louis, MO, USA). All reagents and solvents were commercially provided at HPLC grade. Laboratory animals were supplied by Orient Bio (Seongnam, Korea).

### 2.2. HPLC Analysis

#### 2.2.1. Sample Preparation and Apparatus

The plasma sample (50 µL) with IS was deproteinized with twice the volume of acetonitrile (ACN). All HPLC analyses were performed on an Agilent 1100 series system (Palo Alto, CA, USA). Capcell-pak C_18_ MG120 column (3.0 mm × 250 mm, 5 µm, Shiseido, Tokyo, Japan) was the analytical column used. All the prepared samples were eluted with acetonitrile and water (65:35, *v*/*v*) containing 0.1% trifluoroacetic acid (TFA) at a flow rate of 0.5 mL/min. The sample reservoir and column oven were maintained at 4 °C and 40 °C, respectively. UV detection was performed at 284 nm and the injection volume was 30 µL.

#### 2.2.2. Method Validation

##### Linearity and Sensitivity

The calibration curve of Ewha-18278 was set using eight concentrations ranging between 0.05 and 10 µg/mL (0.05, 0.1, 0.2, 0.5, 1, 2, 5, and 10 µg/mL). The LLOQ was defined as the lowest concentration with a precision of ≤20% coefficient of variation (CV) and an accuracy between 80% and 120%. The LLOQ was no less than the signal-to-noise ratio of 5.

##### Precision and Accuracy

The precision and the accuracy of the analytical method were determined at four quality control (QC) concentration levels (0.05, 0.1, 1, and 5 µg/mL as LLOQ, low, medium and high concentrations). The intra-day precision and accuracy were measured using five sets of QC samples in a day. Inter-day precision and accuracy were determined using a set of QC samples for 5 different days.

##### Recovery and Stability

Extraction recovery was performed using three QC samples (low, medium, and high concentrations) and was determined by dividing the peak area of an extracted sample by the area of a standard solution. The stability of Ewha-18278 in rat plasma was studied by evaluating the short-term (at room temperature and at 37 °C for 6 h, respectively) and long-term (at −20 °C and at −70 °C for 4 weeks, respectively) stabilities, freeze and thaw stability, post-preparative stability and stock solution stability, with low and high QC concentrations. 

### 2.3. Animal Experiments

All the animal procedures were approved by the Institutional Animal Care and Use Committee of Ewha Womans University (IACUC) (No. 2010-2-5, approved on 19 February 2010), Seoul, Korea. Seven-week-old male Sprague-Dawley rats (200–240 g) were used for the PK experiments [7].

### 2.4. PK Studies

#### 2.4.1. Formulations of Ewha-18278

The solutions of 1 mg/mL and 2 mg/mL of Ewha-18278 in two different formulations were prepared immediately prior to intravenous (IV) injection and oral administration (PO), respectively. The DMSO-based formulations were comprised of 10% DMSO and 90% saline-based solution (25% Cremophor^®^ EL, 5% polyethylene glycol 400, 0.5% Tween^®^ and 69.5% saline, *v*/*v*/*v*/*v*). The diazepam injection-based formulation applicable to humans was composed of 40% propylene glycol, 10% dehydrated ethanol, and 50% water (*v*/*v*/*v*). In the case of the diazepam injection-based formulations, Ewha-18278 was prepared in two different concentrations, 1 mg/mL and 2 mg/mL for oral administration. These concentrations were utilized to examine the effect of concentration on the PK parameters of the compound. 

#### 2.4.2. Administrations of Ewha-18278

The rats were divided into five groups on the day of the drug administration. Two groups (*n* = 6) were administered the DMSO-based formulation: IV injection of 2 mg/kg and oral administration of 20 mg/kg. Three groups (*n* = 5–6) were treated with the diazepam injection-based formulation: IV injection of 2 mg/kg and oral administration of 20 mg/kg in two different concentrations (1 mg/mL and 2 mg/mL). The common carotid artery was catheterized for blood sampling. Blood sampling (0.2 mL) was conducted at 0, 0.033, 0.083, 0.25, 0.5, 1, 2, 4, 6, 10, and 24 h for the IV group and 0, 0.083, 0.25, 0.5, 1, 2, 4, 6, 10, 24, and 34 h for PO groups [8,9]. Plasma samples mixed with IS (Ewha-18212) were analyzed by the HPLC system.

#### 2.4.3. Analysis of Ewha-18278 PK Data

WinNonlin^®^ Professional version 5.2 software (Pharsight Corporation, Mountain View, CA, USA) was used to estimate the PK parameters following intravenous or oral administration of Ewha-18278 to rats. Non-compartmental analysis was performed using the plasma Ewha-18278 concentration-time profiles to obtain the following PK parameters: initial plasma concentration (C_0_), area under the plasma concentration-time curve from zero time to infinity (AUC_inf_), elimination half-life (t_1/2_), apparent volume of distribution (V_d_), total clearance (Cl_t_), apparent volume of distribution following oral administration (V_d_/F), and oral clearance (Cl_t_/F). The maximum plasma concentration (C_max_) and the time required to reach C_max_ (T_max_) were directly measured from the plasma Ewha-18278 concentration-time curve. The absolute BA (F, %) of Ewha-18278 was calculated by using the equation:

F = [(AUC_po_/AUC_iv_) × (Dose_iv_/Dose_po_) × 100]

### 2.5. Data Analysis

Statistical analysis was conducted using *t*-test and Dunnett’s test in conjunction with one-way analysis of variance (ANOVA) for the PK studies. Free GraphPad Prism was used for the analysis (Version 8.1.2, La Jolla, CA, USA). Mean data were presented with standard deviation (SD). Statistical significance was indicated by *p*-values < 0.05.

## 3. Results

### 3.1. HPLC Method Validation

The HPLC-UV method for the analysis of Ewha-18278 in rat plasma, was validated according to USA-FDA guidance [10]. Ewha-18278 and IS were eluted at approximately 14.1 min and 15.9 min, respectively, without any interfering peaks. In addition, the calibration curve of Ewha-18278 was linear over the concentrations of 0.05–10 µg/mL with the correlation coefficient (r) of 1.000. The analytical method was found to be valid in terms of specificity, linearity, accuracy, and precision (Table S1). The extraction recoveries of Ewha-18278 and IS were 93.3 ± 6.30–93.8 ± 1.40% and 88.0 ± 1.09%, respectively. The stability results of the compounds were within 85–115% compared to freshly prepared QC samples (Table S2). This validated HPLC-UV analytical method was applied to determine the plasma concentrations of EWHA-18278 following IV and oral administrations of the compound.

### 3.2. Pharmacokinetics of Ewha-18278 in DMSO-based Formulation

**Figure 2:**
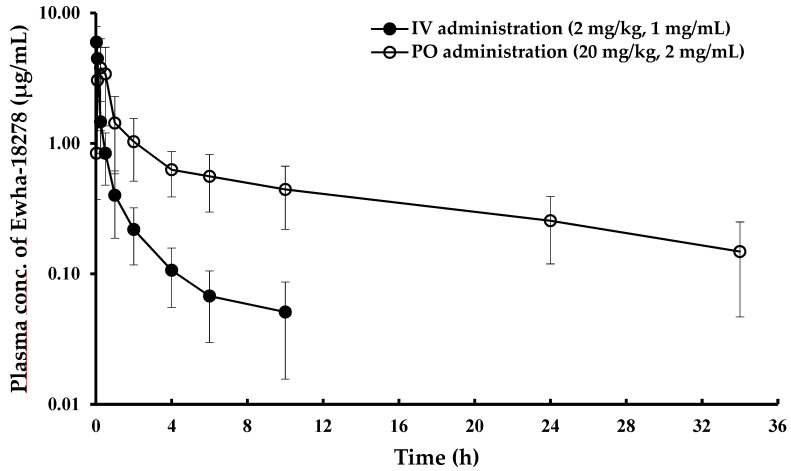
Mean plasma concentration-time profiles of Ewha-18278 in DMSO-based formulation after IV and oral administrations to rats. ●, IV administration (2 mg/kg); ○, oral administration (20 mg/kg). Error bars represent S.D. (*n* = 6).

The NOX inhibitor was highly insoluble in water and was dissolved in a DMSO-based *co*-solvent system (DMSO-based formulation). Table 1 and Figure 2 showed PK parameters and the mean plasma concentration-time profiles of Ewha-18278, respectively, following IV (2 mg/kg) and oral (20 mg/kg) administrations. The AUC_inf_ and t_1/2_ values of the NOX inhibitor were 2.86 ± 1.09 µg·h/mL and 3.07 ± 2.01 h following IV injection, respectively, and 19.4 ± 8.69 µg·h/mL and 14.2 ± 4.76 h after oral administration, respectively (Table 1). The C_0_ and C_max_ values of the NOX inhibitor were 7.24 ± 2.45 µg/mL and 4.68 ± 2.11 µg/mL, respectively. The T_max_ value was 0.249 ± 0.166 h in the oral administration group, suggesting rapid absorption. The absolute BA was calculated to be 62.4%.

### 3.3. Pharmacokinetics of Ewha-18278 in Diazepam Injection-based Formulation Applicable to Humans

For a clinical application of the NOX inhibitor, we tried to find drug formulation applicable to humans, instead of DMSO-based formulation. The NOX inhibitor was dissolved in another *co*-solvent system used for diazepam injection to humans (diazepam injection-based formulation). The AUC_inf_ and t_1/2_ values of the compound were 2.15 ± 0.693 µg·h/mL and 4.07 ± 2.28 h in the IV injection group, respectively, and 9.25 ± 4.42 µg·h/mL and 8.96 ± 2.92 h in the oral administration group, respectively, when the NOX inhibitor was prepared at the same concentrations of the DMSO-based formulation (1 mg/mL for IV injection and 2 mg/mL for oral administrations) (Table 1). The absolute BA of the compound was 43.0%. Although no statistical significance was found between the two formulations in PK parameters such as AUC_inf_, T_max_, and t_1/2_ following IV or oral administration, the PK parameters of the compound appeared to be decreased in the diazepam injection-based formulation compared with DMSO-based formulation after oral administration.

### 3.4. Initial Concentration Effect on Pharmacokinetics of Ewha-18278 in Diazepam Injection-based Formulation Following Oral Administration

In order to identify the optimal condition of the diazepam injection-based formulation for future clinical application, two different concentrations (1 mg/mL and 2 mg/mL) of Ewha-18278 were prepared in the co-solvent system. When rats received low concentration of the compound (1 mg/mL) at a dose of 20 mg/kg, the values of AUC_inf_, t_1/2_ and absolute BA were 14.4 ± 8.71 µg·h/mL, 14.4 ± 3.73 h, and 67.2%, respectively (Table 1). These PK parameters approximately decreased 35% in the higher concentration group (2 mg/mL) of the same formulation. On the other hand, overall PK parameters in the low concentration group (1 mg/mL) of the diazepam injection-based formulation were similar to those of the DMSO-based formulation (2 mg/mL). Although there was no statistical significance in PK parameters, the lower concentration of the diazepam injection-based formulation appeared to be in better condition for human application.

## 4. Discussion

In the beginning stage, we developed a novel lead compound, #1 (Figure S1), for selective inhibition of NOX1 and NOX4 in the treatment of osteoporosis [11]. This compound was found to be effective in vitro; however, the compound was very unstable in plasma at 37 °C. Also, the absolute BA of the compound was only 7.0%, suggesting that oral absorption of the compound was low. Its low absolute BA was probably due to its low stability in biological fluids. Moreover, its NOX inhibitory activity was much less than Ewha-18278 [5]. Therefore, improvement in its biological stability by chemical modification of its structure was necessary in order to obtain favorable PK profiles without changes in its NOX inhibitory activity.

Another follow-up compound, #2 (Figure S1), with an attached propyl group to the core structure, improved plasma stability and PK profiles and resulted in a 7.8-fold increase in BA [11]. However, the aqueous solubility of the follow-up compound was low: the compound would only dissolve in copious amounts of surfactants and DMSO. This formulation violated the FDA guideline and was not applicable to humans. Accordingly, improving the solubility of the compound and discovering a suitable formulation for practical application to humans was necessary for developing a drug candidate for the treatment of osteoporosis.

In order to improve the low solubility of #2, its hydrochloride salt form, Ewha-18278, was prepared. Generally, salt forms of acidic or basic drug are known to offer higher solubility than their corresponding free forms [12]. Besides, salt formation could improve dissolution rates and BA of free forms without chemical structure modification [12,13]. Indeed, salt formation strongly enhanced the solubility of #2, making the formulation dissolvable. This produced a pharmaceutically acceptable new formulation using co-solvents, which avoided the excessive use of surfactants and DMSO. The newly selected formulation was based on a diazepam injection-based formulation containing 40% propylene glycol, 10% dehydrated alcohol, and 50% water [14].

**Figure 3:**
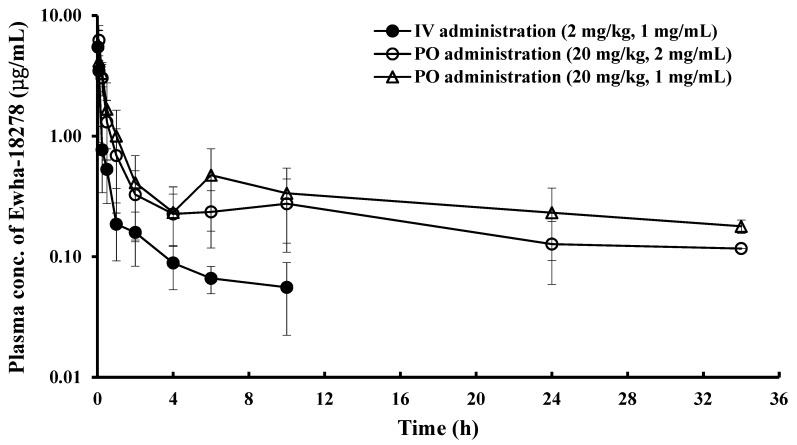
Mean plasma concentration-time profiles of Ewha-18278 in diazepam injection-based formulation after IV (2 mg/kg) and oral (20 mg/kg) administrations to rats. ●, IV administration; ○, oral administration (2 mg/mL); ∆, oral administration (1 mg/mL). Bars represent S.D. (n = 5–6).

In this study, a HPLC/UV method to quantify Ewha-18278 in rat plasma was developed and validated with respect to specificity, linearity, accuracy, and precision. Using this analytical method, plasma concentrations of Ewha-18278 were successfully determined following PK studies. Figure 2 and Figure 3 show the mean plasma concentration-time profiles of Ewha-18278 administered via intravenous and oral routes using DMSO-based and diazepam injection-based formulations. The absorption of the NOX inhibitor was rapid in both formulations, and the BA was more than 40% in all groups. This suggests that the compound has satisfactory permeability in the gastrointestinal (GI) tract. Therefore, this compound can be developed as an oral formulation preferable to both patients and physicians [15].

Although the AUC_inf_ of Ewha-18278 in the DMSO-based formulation was the highest (17.9 µg·h/mL) among three oral dosing groups (Table 1), there were no statistical differences. Several surfactants in the DMSO-based formulation may enhance the membrane fluidity of the intestine by alteration of membrane integrity allowing the compound to easily pass through the intestinal membrane [13,14,16]. However, the DMSO-based formulation cannot be used in clinical approaches because DMSO is restricted for use in humans by the FDA. However, some medications using DMSO in the formulations have been approved recently [17,18]. 

In the diazepam injection-based formulation applicable to human, the AUC_inf_ and BA of the low concentration (1 mg/mL) were higher than those of the high concentration (2 mg/mL) after oral administration. On the other hand, Ewha-18278 was quickly absorbed in the high concentration (Table 1). *Co*-solvent systems are often used to improve the solubility and BA of low water-soluble drugs after oral administration [19]. Ewha-18278 was completely dissolved in *co*-solvents, the mixture of propylene glycol, dehydrated ethanol, and water. In addition, the second peaks of the NOX inhibitor were monitored at 10 h and at 6 h at both high concentration and low concentration, respectively (Figure 3). Although drugs are mostly absorbed in the upper small intestine, some drugs with low solubility may be absorbed in the large intestine [20]; Ewha-18278 may have been absorbed in both the small intestine and large intestine (Figure 3). Also, diluting the drug concentration in *co*-solvents (1 mg/mL) seemed to increase the absorption of the compound in the lower part of GI tract (Figure 3) as compared to high concentration (2 mg/mL). The double volume of *co*-solvents is favorable for keeping the low soluble compound in solution.

## 5. Conclusions

The pharmacokinetics of Ewha-18278 generated for the purpose of osteoporosis therapy were examined in this investigation as a pre-clinical process. The specific NOX1/2/4 inhibitor can be orally delivered to humans because the absolute BA of two formulations was more than 40%. Although the absorption of Ewha-18278 was higher in the DMSO-based formulation (2 mg/mL) than in the diazepam injection-based formulation (1 mg/mL), there was no statistical difference. Therefore, the diazepam injection-based formulation of Ewha-18278 is a suitable candidate for dosage development for future toxicity test and clinical trial.

## Figures and Tables

**Table 1:** Mean pharmacokinetics (PK) parameters of Ewha-18278 following IV (2 mg/kg) and oral (20 mg/kg) administrations to rats.

PK Parameters	DMSO Formulation		Diazepam Injection-Based Formulation		
	IV (1 mg/mL)	PO (2 mg/mL)	IV (1 mg/mL)	PO (2 mg/mL)	PO (1 mg/mL)
C_0_ (µg/mL)	7.24 ± 2.45		7.48 ± 3.42		
C_max_ (µg/mL)		4.68 ± 2.11		6.24 ± 2.02	5.01 ± 2.51
T_max_ (h)		0.249 ± 0.166		0.083 ± 0.00	0.233 ± 0.171
AUC_inf_ (µg·h/mL)	2.86 ± 1.09	19.4 ± 8.69	2.15 ± 0.693	9.25 ± 4.42	14.4 ± 8.17
t_1/2_ (h)	3.07 ± 2.01	14.2 ± 4.76	4.07 ± 2.28	8.96 ± 2.92	14.4 ± 3.73
V_d_ (L)	0.785 ± 0.154		1.52 ± 1.17		
Cl_t_ (L/h)	0.216 ± 0.084		0.250 ± 0.072		
V_d_/F (L)		6.06 ± 2.32		7.90 ± 3.01	9.72 ± 5.17
Cl_t_/F (L/h)		0.327 ± 0.164		0.726 ± 0.553	0.531 ± 0.370
**F (%)**		62.4		43.0	67.2

No significant difference in IV and PO results between DMSO formulation and diazepam injection-based formulation. A part of the PK parameters obtained from diazepam-injection based formulation has been reported previously as supplementary data [5]. Data are presented as mean ± S.D. (*n* = 5–6 rats per group).

## Supplementary Materials

The following are available online at https://www.mdpi.com/1999-4923/11/9/482/s1, Figure S1: The chemical structures of (A) compound #1 and (B) compound #2, Table S1: Intra-day and inter-day accuracy and precision of Ewha-18278 in rat plasma, Table S2: Stability of Ewha-18278 (*n* = 3).

## References

[1] Krause K.H., Bedard K. (2008). NOX enzymes in immuno-inflammatory pathologies. Semin. Immunopathol..

[2] Bedard K., Krause K.H. (2007). The NOX family of ROS-generating NADPH oxidases: Physiology and pathophysiology. Physiol. Rev..

[3] Lee N.K., Choi Y.G., Baik J.Y., Han S.Y., Jeong D.W., Bae Y.S., Kim N., Lee S.Y. (2005). A crucial role for reactive oxygen species in RANKL-induced osteoclast differentiation. Blood.

[4] Valko M., Leibfritz D., Moncol J., Cronin M.T., Mazur M., Telser J. (2007). Free radicals and antioxidants in normal physiological functions and human disease. Int. J. Biochem. Cell. Biol..

[5] Joo J.H., Huh J.E., Lee J.H., Park D.R., Lee Y., Lee S.G., Choi S., Lee H.J., Song S.W., Jeong Y. (2016). A novel pyrazole derivative protects from ovariectomy-induced osteoporosis through the inhibition of NADPH oxidase. Sci. Rep..

[6] Shargal L., Yu A.B.C., Shargal L., Yu A.B.C. (2016). Introduction to biopharmacrutics and pharmacokinetics. Applied Biopharmarceutics and Pharmacokinetics.

[7] Lee J., Chae S.W., Oh A.R., Yoo J.H., Park Choo H.Y., Rhie S.J., Lee H.J. (2019). Effects of Piperazine Derivative on Paclitaxel Pharmacokinetics. Pharmaceutics.

[8] Chae S.W., Woo S., Park J.H., Kwon Y., Na Y., Lee H.J. (2015). Xanthone analogues as potent modulators of intestinal P-glycoprotein. Eur. J. Med. Chem..

[9] Chae S.W., Lee J., Park J.H., Kwon Y., Na Y., Lee H.J. (2018). Intestinal P-glycoprotein inhibitors, benzoxanthone analogues. J. Pharm. Pharmacol..

[10] U.S. Food & Drug. https://www.fda.gov/regulatory-information/search-fda-guidance-documents/bioanalytical-method-validation-guidance-industry.

[11] Oh J.H. (2010). A Simple HPLC Method for Quantification of NOX Inhibitors in Rat Plasma and Its Application to Pharmacokinetic Study. Master’s Thesis.

[12] Serajuddin A.T. (2007). Salt formation to improve drug solubility. Adv. Drug Deliv. Rev..

[13] Morris K.R., Fakes M.G., Thakur A.B., Newman A.W., Singh A.K., Venit J.J., Spagnuolo C.J., Serajuddin A.T. (1994). An integrated approach to the selection of optimal salt form for a new drug candidate. Int. J. Pharm..

[14] Strickley R.G. (2004). Solubilizing excipients in oral and injectable formulations. Pharm. Res..

[15] Mignani S., El Kazzouli S., Bousmina M., Majoral J.P. (2013). Expand classical drug administration ways by emerging routes using dendrimer drug delivery systems: A concise overview. Adv. Drug Deliv. Rev..

[16] Xia W.J., Onyuksel H. (2000). Mechanistic studies on surfactant-induced membrane permeability enhancement. Pharm. Res..

[17] Swanson B.N. (1985). Medical use of dimethyl sulfoxide (DMSO). Rev. Clin. Basic Pharm..

[18] PharmTech. http://www.pharmtech.com/advances-regulated-pharmaceutical-use-dimethyl-sulfoxide-usp-pheur?pageID=1.

[19] Pouton C.W. (2006). Formulation of poorly water-soluble drugs for oral administration: Physicochemical and physiological issues and the lipid formulation classification system. Eur. J. Pharm. Sci..

[20] Park J.H., Park J.H., Hur H.J., Woo J.S., Lee H.J. (2012). Effects of silymarin and formulation on the oral bioavailability of paclitaxel in rats. Eur. J. Pharm. Sci..

