# Supplementary material for: Pharmacokinetic Study of NADPH Oxidase Inhibitor Ewha-18278, a Pyrazole Derivative
Source: Pharmaceutics. 2019 Sep 17;11(9):482. doi: 10.3390/pharmaceutics11090482 (PMC6781499; doi:10.3390/pharmaceutics11090482)
Supplement: Supplementary file 1 [file pharmaceutics-11-00482-s001.pdf]

pharmaceutics

MDPI

Supplementary Materials: Pharmacokinetic Study of NADPH Oxidase Inhibitor Ewha-18278, a Pyrazole Derivative

Seul Gee Lee, Jaeok Lee, Kyung Min Kim, Kee-In Lee, Yun Soo Bae and Hwa Jeong Lee

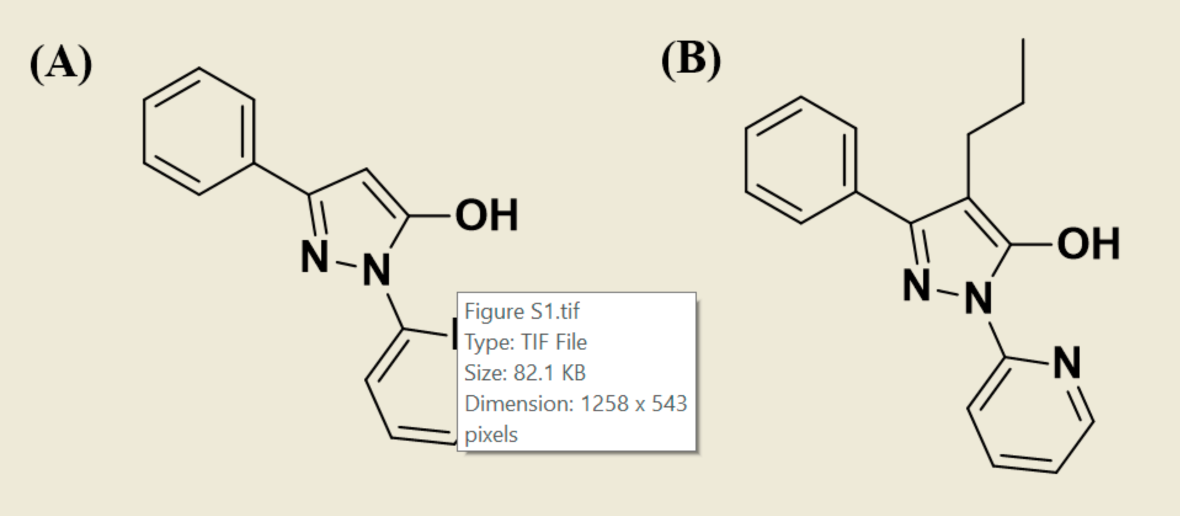

Figure S1. The chemical structures of (**A**) compound #1 and (**B**) compound #2.

Table S1. Intra-day and inter-day accuracy and precision of Ewha-18278 in rat plasma.

Concentration (μg/mL)	Intra-day (*n* = 5)			Inter-day (*n* = 5)		
	Measured Conc.[a] (μg/mL)	Accuracy (%)	Precision (%, CV)	Measured Conc.[a] (μg/mL)	Accuracy (%)	Precision (%, CV)
0.05	0.046 ± 0.008	91.5	16.4	0.046 ± 0.005	92.5	9.9
0.1	0.095 ± 0.010	94.7	10.6	0.097 ± 0.007	98.5	7.4
1	0.954 ± 0.052	95.4	5.5	1.018 ± 0.067	102.3	6.6
5	5.061 ± 0.135	101.2	2.7	5.201 ± 0.166	104.2	3.2

[a], Mean ± SD.

Table S2. Stability of Ewha-18278 (*n* = 3).

Compound (Conc.)	RT, 6 h (%)	37 °C, 6 h (%)	−20 °C, 4-W (%)	−70 °C, 4-W (%)	Freeze-thaw (%)	Post-prep. (%)
Low	94.5	89.0	99.4	100	90.4	103
High	93.4	87.5	90.4	97.5	86.0	103

Conc., concentration; h, hour; W, week.
